# Patient-reported outcomes of laser-assisted pain control following non-surgical and surgical periodontal therapy: A systematic review and meta-analysis

**DOI:** 10.1371/journal.pone.0238659

**Published:** 2020-09-17

**Authors:** Risako Mikami, Koji Mizutani, Yoshiyuki Sasaki, Takanori Iwata, Akira Aoki

**Affiliations:** 1 Department of Periodontology, Graduate School of Medical and Dental Sciences, Tokyo Medical and Dental University (TMDU), Bunkyo, Tokyo, Japan; 2 Department of Maxillofacial Surgery, Graduate School of Medical and Dental Sciences, Tokyo Medical and Dental University (TMDU), Bunkyo, Tokyo, Japan; Massachusetts General Hospital, UNITED STATES

## Abstract

Adjunctive use of laser devices as high reactive-level laser/light therapy (HLLT) or photobiomodulation therapy (PBMT) for periodontal therapy is known to be more effective on suppressing pain than conventional therapy, however, there are no systematic reviews addressed its effectiveness. This systematic review and meta-analysis aim to investigate the following clinical question (CQ): does adjunctive use of lasers with conventional therapy suppress the pain associated with periodontal treatment? A systematic and extensive literature search was performed to summarize the currently available knowledge to answer the CQ using the PubMed, Cochrane Library, and Web of Science databases for randomized controlled trials (RCTs) conducted before June 2020. Bias risk was assessed using the Cochrane tool for the risk of bias evaluation. A meta-analysis was performed on quantitative evaluation of pain control based on patient-reported outcomes. After an independent screening of 165 initial records, ten RCTs were included. Six of them focused on surgical procedures and the others on non-surgical periodontal pocket therapy. The protocols of HLLT, PBMT, and combination with HLLT and PBMT were employed in five, four and one RCTs, respectively. Following the assessment of bias risk, it is revealed that all RCTs had methodological weaknesses regarding the blinding of key personnel, although other bias risk factors were not evident. Meta-analysis showed that HLLT using erbium lasers significantly reduced the patient-reported pain immediately after treatment (two RCTs, p < 0.0001), while PBMT using diode lasers significantly reduced pain 2–7 days after treatment (two RCTs, p < 0.0001 to p = 0.03). The presented systematic review and meta-analysis suggest that the alternative use of HLLT using erbium lasers to conventional instrumentation can significantly suppress postoperative pain and that intraoperative or postoperative PBMT using diode lasers combined with periodontal surgery can significantly reduce postoperative pain. However, the evidence is still insufficient and more well-designed RCTs are required.

## Introduction

Lasers have been used in various dental treatments, such as soft tissue management, bone and teeth cutting, calculus removal, and promotion of wound healing. In periodontics, lasers therapy is mainly used in conjunction with conventional modalities. The use of lasers is divided into two modalities: high reactive-level laser/light therapy (HLLT), which is utilized for incision and tissue ablation or debridement, and low reactive-level laser/light therapy (LLLT), which aims to promote the postoperative wound healing of the surrounding tissue [1–4], and has recently been expressed alternatively by “photobiomodulation therapy (PBMT)” [5, 6]. There are some controversial reports on whether HLLT or PBMT could be clinically effective [7, 8] or only have few adjunctive effects [9–11] on periodontal therapy. Several systematic reviews have been published, and they have pointed out the need for further studies to clarify the clinical benefits of adjunct laser therapy [9–11].

Recently, a consensus report by the American Academy of Periodontology suggested the necessity of patient-reported outcomes, such as pain, esthetics, and overall patient satisfaction, for optimal treatment strategies [12]. Regarding the effect of lasers on pain control, it is reported that periodontal laser therapy reduced intraoperative and postoperative pain. Currently, there are no systematic reviews addressing the effectiveness of pain control with HLLT or PBMT. Therefore, this study aimed to conduct a systematic review and meta-analysis investigating the following clinical question (CQ): Does adjunctive use of lasers with conventional therapy suppress the pain associated with periodontal treatment?

## Materials and methods

A search strategy was applied according to the Preferred Reporting Items for Systematic Reviews and Meta-analysis (PRISMA) protocol (S1 File) [13, 14].

### Search strategy

An extensive literature search was performed to summarize the currently available knowledge to answer the CQ using the PubMed, Cochrane Library and Web of Science databases for randomized controlled trials (RCTs) investigating the effect of laser irradiation on pain associated with periodontal treatment prior to June 20th, 2020. The search terms used in PubMed are listed below: ("periodontitis"[MeSH Terms] OR "periodontitis"[All Fields] OR "periodontal diseases"[MeSH Terms] OR ("periodontal"[All Fields] AND "diseases"[All Fields]) OR ("periodontal"[All Fields] AND "disease"[All Fields]) OR "Chronic periodontitis"[Mesh Terms] OR ("Chronic"[All fields] AND "periodontitis"[All fields]) OR "gingival recession"[MeSH Terms] OR ("gingival recession"[All fields]) OR "free gingival graft"[All fields] OR "connective tissue graft"[All fields]) AND ("lasers"[MeSH Terms] OR "lasers"[All Fields] OR "laser therapy"[MeSH Terms] OR ("laser"[All Fields] AND "therapy"[All Fields]) OR "low-level light therapy"[MeSH Terms] OR ("low-level"[All Fields] AND "light"[All Fields] AND "therapy"[All Fields]) OR "laser therapy, low level "[All Fields] OR photobiomodulation[All Fields]) AND (("pain"[All Fields] AND ("measure"[All Fields] OR "measuring"[All Fields] OR "measurement"[All Fields])) OR "pain measurement"[MeSH Terms] OR "Pain, Postoperative"[MeSH Terms] OR ("postoperative"[All Fields] AND "pain"[All Fields]) OR "Pain Management" [MeSH Terms] OR ("Pain"[All Fields] AND ("management"[All Fields] OR "managing"[All Fields] OR "control"[All Fields] OR "controlling"[All Fields]))). A similar search strategy was applied in all databases. Additional electronic search was performed in the *Journal of Periodontology*, *Journal of Clinical Periodontology*, *Journal of Periodontal Research*, *Lasers in Surgery and Medicine*, and *Lasers in Medical Science*.

### Study selection

In the first stage, titles and abstracts of all retrieved articles were screened for potentially eligible studies. Full-length articles of the previously identified studies were examined in detail according to eligibility criteria for inclusion in this review. Two reviewers (RM, KM) independently performed the screening process. When there was a disagreement between reviewers, a consensus was reached through further discussions.

The following inclusion criteria were applied:

1. RCTs examining the efficacy of laser therapy (HLLT or PBMT) on pain associated with periodontal therapy.
2. Participants received periodontal treatment with non-surgical therapy, such as scaling and root planing, or surgical therapies, such as flap procedure and periodontal plastic surgery.
3. Participants were allocated to an experimental or placebo/control group.
4. Outcome variables included prevalence, time course and intensity of pain
5. Literature published in English.

The following exclusion criteria were applied:

1. Review articles, case reports, descriptive studies, opinion articles, abstracts, animal experiments or *in vitro* studies.
2. Any studies including photodynamic therapy involving laser irradiation in conjunction with a photosensitizer.

### Assessment of risk of bias

Risk of bias was evaluated in accordance with the Cochrane Handbook for Systematic Reviews of interventions, using the following parameters: adequacy of sequence generation; allocation concealment; blinding of participants, personnel and outcome assessors; incomplete outcome data; and selective outcome reporting [14].

### Data extraction

Data that contained fundamental information and outcomes, including publication information, country, study design, sample size, participant characteristics, randomization method, allocation concealment, blinding measures, intervention and placebo or control approach, laser parameters and regimen, outcome measurements, follow-up duration, patients lost to follow-up, and the occurrence of any adverse events were collected.

### Statistical analysis

The weight of each individual study included in the meta-analysis was determined by its reported standard deviation and sample size. The effect size was estimated and reported as the mean difference (MD) with the 95% confidence interval (CI) for visual analogue scale (VAS) score. Since the mechanism of action depends on the type of lasers, each type of lasers (diode lasers, Nd:YAG lasers and erbium lasers) was analyzed as different subsets. Because each analysis included a small number of studies, the variance between studies was poorly estimated. Thus, a fixed-effect model was adopted for all analyses [13]. Heterogeneity was assessed with a chi-square test and the I^2^ statistic at an alpha level of 0.10. The meta-analysis was performed using REVMAN 5.3. The statistical significance level for the hypothesis test was set at an alpha level of 0.05 for two-tailed z tests.

## Results

### Search and selection results

**Fig 1:**
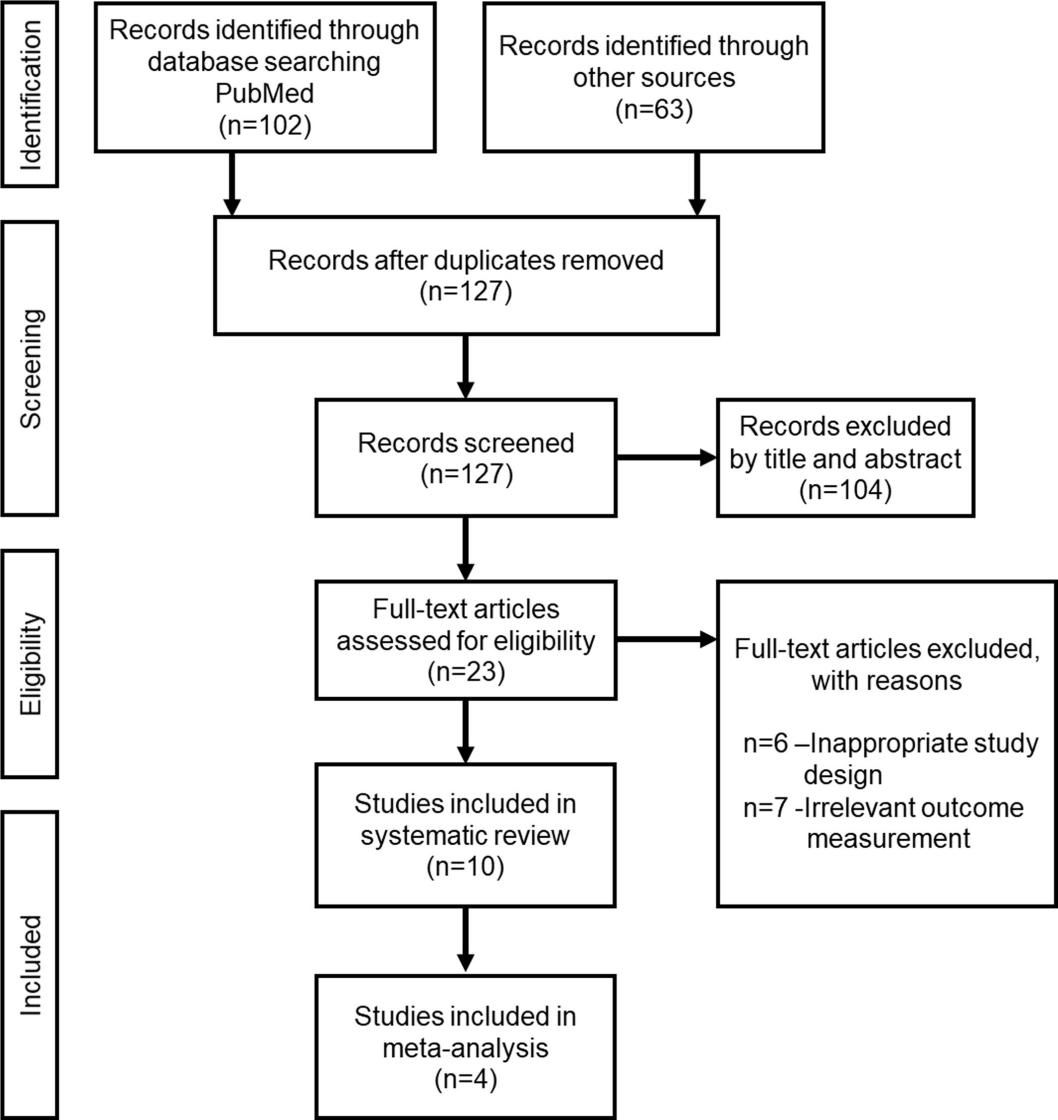
PRISMA flow diagram of the study inclusion criteria.

The study selection process is shown in Fig 1. Overall, 165 studies were identified after the initial search; after removing duplicates, 127 studies were included. During the first stage, 104 studies were excluded based on the evaluation of titles and abstracts (inter-reviewer agreement, kappa statistic = 0.92). Second, after screening the full-text articles of the remaining 23 studies, 13 studies were excluded because of inappropriate study design [15–20] and irrelevant outcome measurement [21–27] (S1 Table) and 10 eligible studies [28–37] (five for HLLT, four for PBMT, and one for both HLLT and PBMT) were included in this systematic review (inter-reviewer agreement, kappa statistic = 0.91). Of these, four studies [29, 31, 33, 34], which showed adequate continuous data concerning the pain level measured with VAS score, were included in the meta-analysis.

### Characteristics of included studies

Study characteristics and laser parameters are shown in Tables 1 and 2, respectively. Five of ten RCTs [28–30, 36] employed the HLLT protocol, four [32–35] used the PBMT protocol, and one [37] used both the HLLT and PBMT protocol. These studies were conducted in seven countries, with sample sizes of 13–40, and the mean age of participants was 25.5 ± 6.3 to 55.3 ± 10.0 years old. Three publications [30, 34, 35] performed parallel design, while the remaining did split-mouth design. While not described in one study [28], all participants in the other publications did not suffer from diseases known to affect the healing following periodontal therapy such as diabetes. In three articles [30, 34, 35] that root coverage was conducted, participants had gingival recession but were otherwise healthy. In the remaining articles, chronic periodontitis patients were recruited. Smokers were excluded in all but three studies [29, 36, 37]. No study reported side effects associated with laser irradiation.

**Table 1:** Characteristics of the included studies.

Study ID	Year	Number of participants (Male/Female)	Age in mean ± SD (range)	Country	Center	Category	Grouping methods	Periodontal treatment	Study design	Evaluation interval	Main findings
Ozcelik *et al*. [32]	2008	22 (12/10)	N/A (31-49)	Turkey	University hospital	PBMT	1. EMD, 2. EMD+PBMT	Regenerative therapy (EMD)	Split-mouth	Day 1-7	EMD+PBMT had resulted in less gingival recession, less swelling and less VAS scores compared with EMD alone.
Braun *et al*. [28]	2010	40 (21/19)	55.3 ± 10.0	Germany	University hospital	HLLT	1. Laser, 2. Sc	Sc	Split-mouth	Post-treatment	Pain assessment showed that laser treatment caused less pain than the sonic device with no difference in the treatment time.
Rotundo *et al*. [29]	2010	27 (9/18)	50.5 ± 11.7	Sweden	University hospital	HLLT	1. Sc, 2. Laser+SRP, 3. Laser, 4. SRP	SRP	Split-mouth	Pre- and post-treatment, 1w, 6 M	The adjunctive use of laser to SRP did not show additional effectiveness in periodontal condition. Laser+SRP group tended to have less pain immediately and one week after treatment than SRP alone group, however the differences are not significant.
Slot *et al*. [36]	2012	30 (13/17)	48.7 ± 11.3 (39-65)	Netherlands	Private clinic	HLLT	1. SRP, 2. Laser+SRP	SRP	Split-mouth	Post-treatment	The adjunctive use of laser to SRP did not show additional effectiveness in periodontal condition. Laser+SRP treated quadrants presented with significantly more postoperative pain.
Sanz-Moliner *et al*. [37]	2013	13 (8/5)	52 ± 8.5	USA	University hospital	HLLT and PBMT	1. Fop, 2. Fop+Laser	Fop	Split-mouth	Day 1-7	Statistically significant differences were shown for pain scale assessment and pain medication consumption favoring test sites.
Yilmaz *et al*. [30]	2014	32 (13/19)	Test: 29.0 ± 4.1, Control: 29.3 ± 4.8	Turkey	University hospital	HLLT	1. Laser-assisted laterally positioned flap, 2. Laterally positioned flap	Laterally positioned flap	Parallel	1 w	The ratio of complete root coverage in test group is significantly higher than control group. There were no differences in VAS pain score between the groups.
Ge *et al*. [31]	2017	31 (14/17)	35.2 ± 9.8 (24-72)	China	University hospital	HLLT	1. Laser, 2. SRP	SRP	Split-mouth	Post-treatment	The reduction of PPD and BOP at weeks 6 and 12 was significantly higher in laser group than in SRP group. The VAS pain score was significantly lower in laser group than in SRP group.
Heidari *et al*. [33]	2018	30 (14/16)	42.5 ± 9.5 (23-63)	Iran	Private clinic	PBMT	1. Fop+PBMT, 2. Fop	Fop	Split-mouth	Day 1-7	Patients reported less pain on days 2, 3, 4, 5, 6, and 7 after surgery in Fop+PBMT group. Furthermore, fewer analgesics were used in this group on days 3, 4, 5, 6, and 7 following the surgery.
Yildiz *et al*. [34]	2018	30 (2/28)	Test: 25.53 ± 6.25, Control: 26.94 ± 7.06	Turkey	University hospital	PBMT	1. FGG+PBMT, 2. FGG	FGG	Parallel	Day 1-7	FGG+PBMT group showed significantly lower VAS pain score and less number of analgesic used.
Isler *et al*. [35]	2018	36 (12/24)	Test: 41.25 ± 10.91, Control: 38.41 ± 14.66	Turkey	University hospital	PBMT	1. PBMT, 2. Ozone, 3. Control	FGG	Parallel	Day 1, 2, 3, 7, 14, 30	No significant difference was obsereved in each group regarding the remaining wound area. Regarding VAS pain score, the control group had higher VAS scores at all time points, no statistically significant difference was observed between the groups.

HLLT, high reactive-level laser/light therapy; Sc, Scaling; SRP, scaling and root planing; VAS, visual analogue scale; PPD, probing pocket depth; BOP bleeding on probing; PBMT, photobiomodulation therapy; EMD, enamel matrix derivative; Fop, flap operation; FGG, free gingival graft technique

**Table 2:** Parameters and regimens of lasers applied in the included studies.

Study ID	Year	Category	Type of laser	Wavelength (nm)	Output power	Repetition rate	Method of application
Ozcelik *et al*. [32]	2008	PBMT	Diode	588	4 J/cm^2^	N/A	Immediately after EMD application, the defect area was irradiated with PBMT for 10 min. PBMT was applied from the outer buccal and lingual surfaces for 5 min each, immediately after suturing, and daily for 5 days.
Braun *et al*. [28]	2010	HLLT	Er:YAG	N/A	120 mJ/pulse (panel)	10 Hz	The laser was used to remove subgingival biofilm. Maximum irradiation time was set to 2 min per tooth.
Rotundo *et al*. [29]	2010	HLLT	Er:YAG	2,940	150 mJ/pulse	10 Hz	The application of the laser treatment was performed from coronal to apical direction with an inclination of the conic fibre tip of about 20° under water irrigation.
Slot *et al*. [36]	2012	HLLT	Nd:YAG	1,064	400 mJ/pulse (panel)	50 Hz	The fibre tip was held in contact with the tissue and aligned parallel to the tooth with water cooling. The laser was applied for no more than 60 sec per site.
Sanz-Moliner *et al*. [37]	2013	HLLT and PBMT	Diode	810 ± 20	1 W (HLLT) 4 J/cm^2^ (PBMT)	CW	HLLT was performed to remove all visible epithelium in the inner side of the flap from the free gingival margin to the bottom of the apical aspect of the flap. For PBMT, all surfaces of the flap, inner and outer, exposed bone, and exposed root structures involved in the surgery were irradiated, leading to a total dosage of 4 J/cm^2^ per surface.
Yilmaz *et al*. [30]	2014	HLLT	Diode	810	3 W	CW	An external horizontal releasing incision on the vestibular alveolar mucosa and de-epithelialization of the interdental papilla were performed with diode laser.
Ge *et al*. [31]	2017	HLLT	Er,Cr:YSGG	2,780	1.25 W	30 Hz	The fiber optic head was moved around the periodontal pockets and across the surface of the root furcations, while debris was washed out of the priodontal pockets under water irrigation.
Heidari *et al*. [33]	2018	PBMT	Diode	940	40 J/cm^2^	CW	PBMT ware applied to both buccal and lingual surfaces with energy density 40 J/cm^2^ and the irradiated area was 2.8 cm^2^. PBMT were performed only during surgery.
Yildiz *et al*. [34]	2018	PBMT	Diode	810	6 J/cm^2^	CW	PBMT was applied with output power 0.1 W for 60 sec at recipient sites immediately, 1, 3, 7, and 14 days after surgery.
Isler *et al*. [35]	2018	PBMT	Diode	970 ± 15	2 W, 5.25 J/cm^2^	CW	Total irradiation time was 30 sec (6 sec for each point). PBMT was performed immediately, 1, 3 and 7 days after surgery.

HLLT, high reactive-level laser/light therapy; CW, continuous wave; PBMT, photobiomodulation therapy; EMD, enamel matrix derivative

Four [28, 29, 31, 36] of six HLLT studies focused on laser-assisted non-surgical periodontal pocket therapy, and two [30, 37] included a surgical periodontal procedure. Regarding PBMT, all studies performed the irradiation during or after periodontal surgical therapy. The following types of lasers were used in HLLT studies; two Er:YAG (2,940 nm) studies, one Er,Cr:YSGG (2,780 nm) study, two diode (810 nm) studies and one Nd:YAG study (1,064 nm) and. In all PBMT studies, diode lasers (588–970 ± 15 nm) were used. The output power of laser irradiation ranged from 1 to 3 W and from 120 to 400 mJ in HLLT, and 4 to 40 J/cm^2^ in PBMT. Two studies [33, 37] employed a single irradiation protocol and multiple irradiation protocols were employed in three PBMT studies [32, 34, 35].

Parameters related to pain were set as the primary outcome in three studies [28, 33, 37] and as the secondary outcome in the remaining studies; in the latter, clinical parameters were used as the primary outcome. Eight of ten included studies used VAS score to measure pain intensity. To perform the meta-analysis, we converted the VAS values from all studies from 0 to 100, while some studies used VAS from 0 to 10. Observational periods about pain ranged from immediately after surgery [28, 29, 31, 36] to six months [29] in HLLT studies, and from one week [32–34] to 30 days [35] in PBMT studies.

Two of the HLLT studies showed significant pain control after using lasers. Braun *et al*. [28] compared the group using sonic scaling with the group using Er:YAG laser for scaling during supportive periodontal therapy (SPT) and showed that the Er:YAG laser group had significantly less pain immediately after treatment. Ge *et al*. [31] compared the Er,Cr:YSGG laser-treated group and the hand instrument-treated group for non-surgical periodontal therapy for furcation involvement, and less pain was shown in the Er,Cr:YSGG-treated group immediately after treatment. Rotundo *et al*. [29] compared the effects of non-surgical periodontal pocket treatment in the following four groups, using the Er:YAG laser: 1) scaling group, 2) laser+scaling root planing (SRP) group, 3) laser group, 4) SRP group. Although they showed no significant differences between groups in probing pocket depth (PPD), bleeding on probing, clinical attachment level (CAL) gain, gingival recession and plaque index six months after treatment, the laser group showed a tendency for less pain immediately and one week after treatment, compared to the SRP group. Conversely, Yilmaz *et al*. [30] showed no significant difference in VAS score for pain one week after laterally positioned flap operation between the diode laser-treated group and the scalpel-treated group for vestibular deepening incision. Slot *et al*. [36] showed that the adjunctive use of Nd:YAG laser to SRP presented with significantly more postoperative pain than SRP alone by the questionnaire about pain.

Three of the PBMT studies showed efficient pain control after using lasers. Ozcelik *et al*. [32] used a diode laser for PBMT in periodontal regenerative therapy using enamel matrix derivative and reported that PBMT groups showed significantly less pain one and two days after surgery compared to the non-PBMT group. Heidari *et al*. [33] compared pain between PBMT and non-PBMT groups that used a diode laser in flap operation. The PBMT group showed significantly less pain between days 2 and 7 after surgery. Yildiz *et al*. performed PBMT using a diode laser at the recipient site during free gingival graft (FGG), and they showed that the average value of VAS score for pain between days one and seven after surgery was significantly lower in the PBMT group than in the non-PBMT group. On the other hand, Isler *et al*. [35] performed PBMT using a diode laser to the donor site after FGG and the PBMT group tended to have lower VAS score for pain, but no significant difference was reported between one and seven days after treatment. Sanz-Moliner *et al*. [37] used a diode laser on periodontal flap surgery to de-epithelialize the inner part of the periodontal flap as HLLT and photo-biostimulate the surgical area as PBMT and showed that statistically significant differences favoring test sites in pain scale assessment and pain medication consumption.

### Assessment of methodological quality

**Fig 2:**
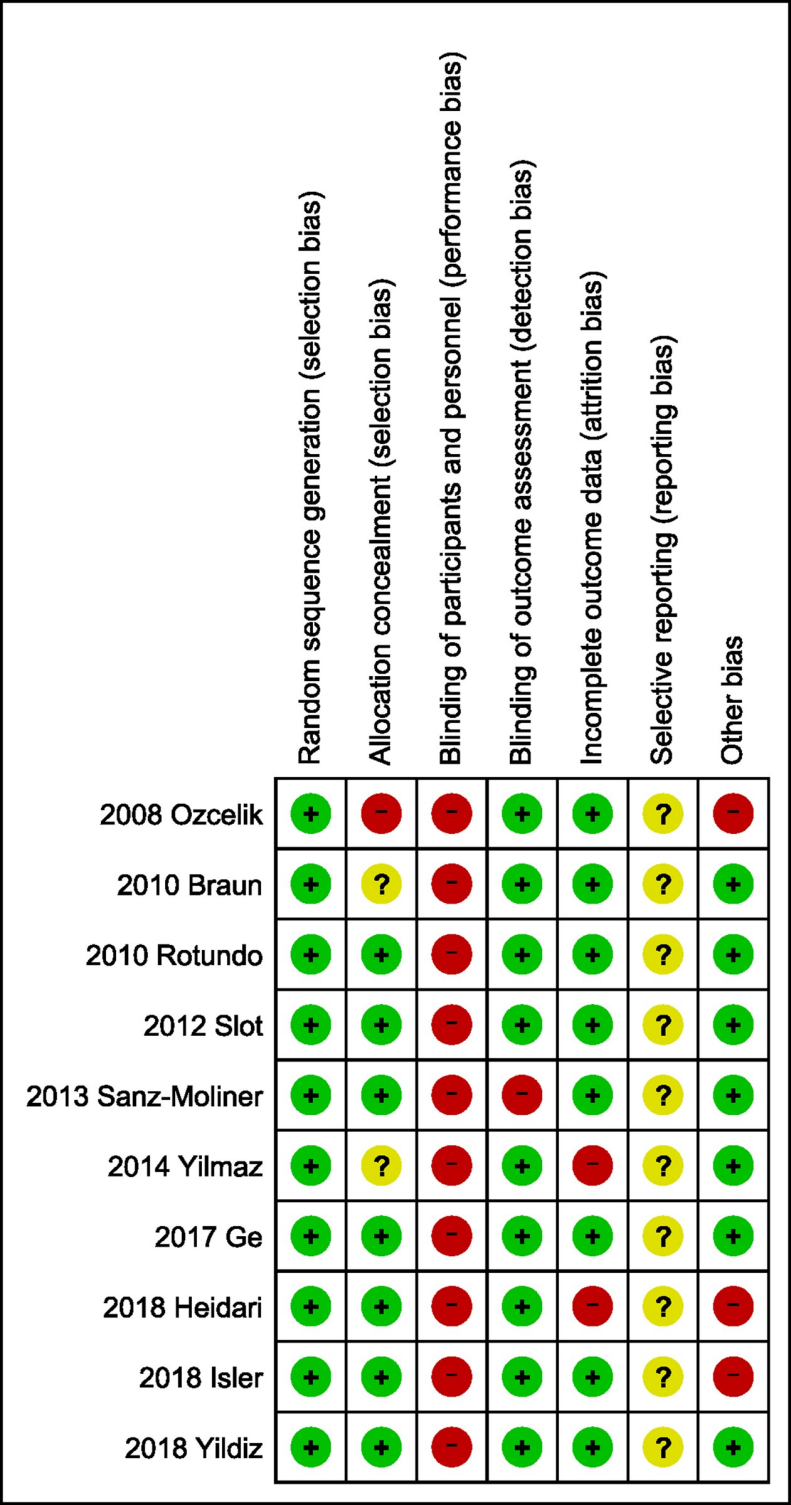
Risk of bias summary: Authors’ judgements about each risk of bias item for each included study.

**Fig 3:**
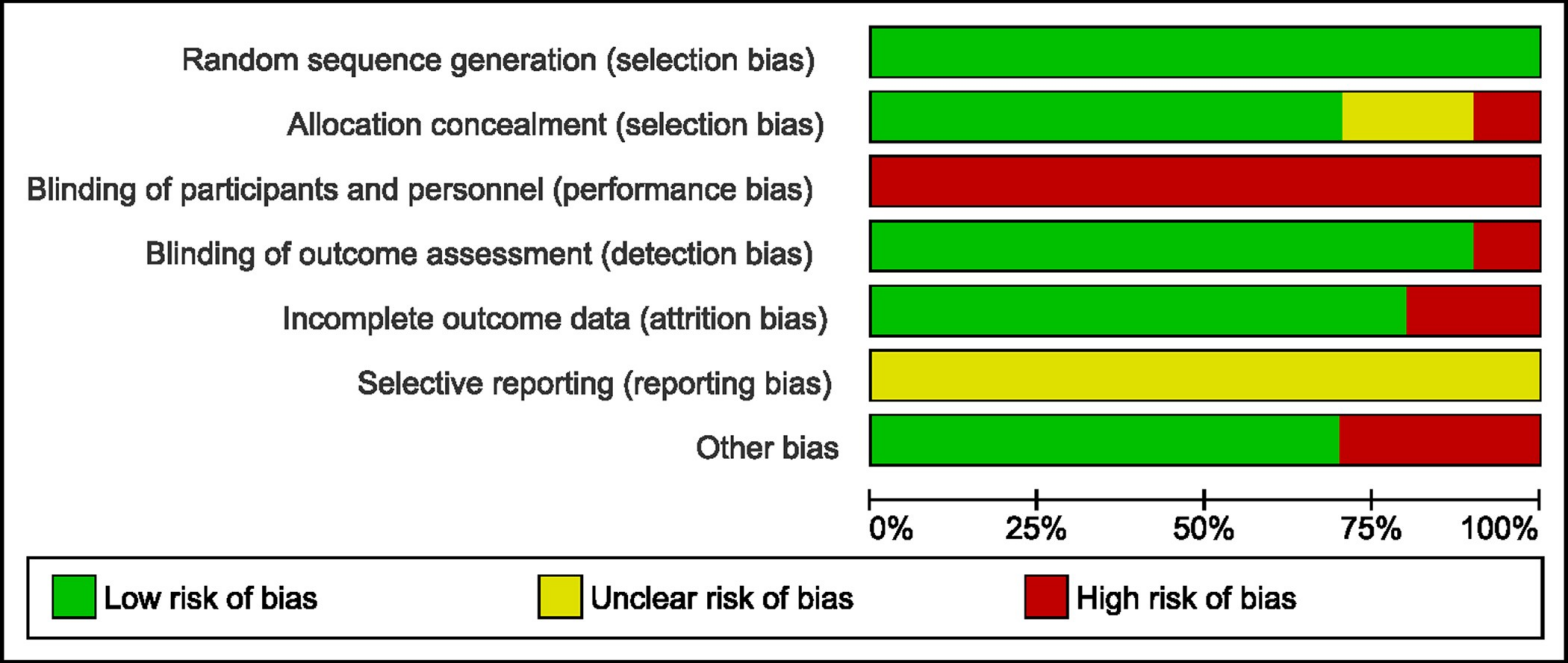
Risk of bias graph: Authors’ judgements about each risk of bias item presented as percentages across all included studies.

The results of the methodological quality assessment are shown in Figs 2 and 3. As shown in Fig 2, all studies were assessed as having a high risk of bias, although they were presented as RCTs. The most commonly used randomization methods were based on computer programs [28–31, 35–36]. Randomization methods included: selection of a sealed envelope (one study) [34], toss of coin (two studies) [32, 37], and random number tables (one study) [33]. Among all seven domains, “blinding of key personnel” accounted for the principal risk factor affecting methodology quality (Fig 3). Three studies were double-blinded for patients and evaluators [32–34] whereas the majority of studies applied a single-blinded method, in which the participant was blinded and the operator, who performed the intervention, was aware of the grouping information. In two studies [34, 36], some patients were lost to follow-up without appropriate explanation or management. There was insufficient information to assess whether outcomes were selectively reported in any of the included studies.

### Effect of laser irradiation on pain associated with periodontal treatment

Adequate continuous data concerning the pain level measured with VAS score was available in four studies [29, 31, 33, 34]. A meta-analysis was conducted to assess the pain suppression effect of HLLT (Table 3) and PBMT (Table 4). Two studies about HLLT using erbium lasers reported the VAS score immediately after treatment and two studies about PBMT using diode lasers reported the VAS score within seven days after treatment. As for HLLT, the laser group showed significantly less VAS score than the control group immediately after treatment (MD, -34.38; 95% CI, -41.47 to -31.13; p < 0.0001). As shown in Table 4, there was no significant difference in VAS score one day after treatment between the control and the PBMT combination groups (MD, -3.10; 95% CI, -12.21 to 6.00; p = 0.50), while between two and seven days after treatment, the PBMT combination group showed significantly less VAS scores than the control group (Table 4).

**Table 3:** Meta-analysis of HLLT effects, comparison: HLLT using Erbium lasers versus conventional instrumentation in periodontal therapy, outcome: VAS score for pain assessment.

				Test for total effect			Test for heterogenity	
	Studies	Number of participants	Model	MD	95% CI	*P* value	*I*^*2*^ value (%)	*P* value
Immediately after treatment	29, 31	114	Fixed	-34.38	-39.33 to -29.44	<0.0001*	84	0.01

MD, mean difference; CI, confidence interval

*p < 0.05, significant difference between HLLT and conventional instrument in periodontal therapy

**Table 4:** Meta-analysis of PBMT effects, comparison: PBMT using diode lasers versus non-PBMT in periodontal therapy, outcome: VAS score for pain assessment.

				Test for total effect			Test for heterogenity	
	Studies	Number of participants	Model	MD	95% CI	*P* value	*I*^*2*^ value (%)	*P* value
Day 1	33, 34	82	Fixed	-3.10	-12.21 to 6.00	0.5	0	0.8
Day 2	33, 34	82	Fixed	-8.67	-16.52 to -0.83	0.03*	0	0.42
Day 3	33, 34	82	Fixed	-14.92	-23.38 to -6.45	0.0006*	7	0.3
Day 4	33, 34	82	Fixed	-13.71	-20.22 to -7.20	<0.0001*	52	0.15
Day 5	33, 34	82	Fixed	-11.09	-17.23 to -4.96	0.0004*	0	0.63
Day 6	33, 34	82	Fixed	-10.82	-17.33 to -4.31	0.001*	0	0.64
Day 7	33, 34	82	Fixed	-7.31	-12.68 to -1.93	0.008*	0	1

MD, mean difference; CI, confidence interval

*p < 0.05, significant difference between PBMT and non-PBMT in periodontal therapy

## Discussion

In this systematic review, we investigated the association between laser irradiation and pain control after periodontal treatment. Based on our systematic review of ten RCTs, we provide evidence showing that laser irradiation combined with periodontal therapy does suppress pain. Our meta-analysis revealed that HLLT significantly suppressed pain immediately after periodontal treatment. In the meta-analysis of HLLT, we compared conventional therapy and HLLT as an alternative modality, because the effects of laser irradiation could not be accurately evaluated in combined therapy. Er,Cr:YSGG laser and Er:YAG laser are included in the meta-analysis because their wavelengths are close and the reactions in the lased tissue are similar, however, the heterogeneity is significant in this meta-analysis and careful interpretation is required. With regard to the meta-analysis of PBMT, periodontal treatment combined with PBMT significantly reduced pain between two and seven postoperative days, but no significant difference was reported one day after treatment. In this meta-analysis, no significant heterogeneity was found.

Of the ten articles included in this systematic review, seven articles additionally examined the effects of laser use on clinical parameters. Four of them [30–32, 34] reported significant differences in periodontal parameters, such as PPD, CAL, ratio of root coverage, gingival recession, and graft shrinkage, while the others reported no significant differences [29, 35, 37]. It is suggested that the beneficial effects of adjunctive laser therapy on periodontal parameters were controversial because of heterogeneous treatment modalities. However, since no study has reported any side effects associated with the use of lasers, the safety of laser application was indicated in the applied energy settings of the above studies.

For HLLT, one of the factors contributing to pain control is that debridement with lasers causes lesser damage to the tissue as compared to conventional instruments. Some *in vivo* and clinical studies showed that incision using lasers is less likely to cause pain compared with incision using a scalpel [38, 39]. Qu *et al*. [40] showed that incision with Er:YAG lasers in rats was less likely to trigger an inflammatory response and caused lesser damage to mucoperiosteal tissue as compared to a scalpel incision. Furthermore, it is known that lasers have a bactericidal effect [16, 41–43] and eliminate lipopolysaccharide [44], thus potentially leading to suppression of the immune responses and inflammation.

The following factors contributed to pain control by PBMT. First, PBMT is known to have an analgesic action. Kasai *et al*. [45] reported an inhibitory effect of PBMT on impulse conduction within a peripheral nerve, and it has been shown that PBMT suppressed the production of substances associated with pain, such as histamine or bradykinin [46]. Moreover, the secretion of inflammatory cytokines is suppressed by PBMT [47, 48], and Zeredo *et al*. [49] showed that PBMT has a tonic antinociceptive effect on inflammatory pain even when applied prior to tissue injury. As for clinical research, Caldrin *et al*. showed that interleukin-1β and tumor necrosis factor-α levels were significantly lower in the group that underwent PBMT combined with SRP than in the SRP alone group [50]. It has been suggested that PBMT suppresses oxidative stress associated with tissue damage since oxidative stress is known to be involved in the wound healing process of the periodontal tissue [51]. Swerts *et al*. showed that PBMT combined with SRP suppressed oxidative stress in experimentally-induced periodontitis of simvastatin-modified rats [52]. Using lasers as HLLT, the minute laser light scattered in the surrounding tissue is assumed to have a beneficial effect as the PBMT. Therefore, HLLT simultaneously accompanies an effect of PBMT, which contributes to pain suppression.

Several limitations of this study should be acknowledged. First, it is desirable to compare by laser type or irradiation protocol because the effect of laser irradiation on periodontal tissue will differ depending on the type of laser or irradiation protocol such as output or repetition rate. However, in this study, because the number of available articles was limited, we discussed without categorizing by type of lasers. Second, although acceptable, all studies included in this systematic review had methodological weaknesses. Third, the number of available studies was insufficient to assert the efficacy of laser-assisted periodontal treatment on pain control. Therefore, it is required to interpret the results of this study carefully. Fourth, we limited our search to articles written in English in this study because most of international peer-reviewed scientific journals accept English as an official language. The inclusion criteria of language might produce language selection bias.

To accumulate evidence regarding the effect of laser treatment, more well-designed RCTs with sufficient sample sizes based on the power calculation should be conducted with reference to Cochrane’s risk of bias assessment criteria. As for the methods of pain assessment, eight of ten studies used the VAS score to evaluate pain in this study. Evaluation of pain is a rather subjective outcome. Pain evaluation will be better reproducible if the methods used are more objective and quantitative. In the future, the effect of laser irradiation in periodontal treatment will become more apparent if well-designed clinical trials are performed and the protocols for laser irradiation are established, which is expected to contribute to the reduction of pain and stress during periodontal treatment.

## Conclusion

The presented systematic review and meta-analysis suggest that the use of HLLT as an alternative to conventional instrumentation can significantly control postoperative pain more effectively compared to conventional periodontal treatment, and intraoperative or postoperative PBMT combined with surgical periodontal therapy can significantly suppress pain. However, careful interpretation of the presented results is required because of the substantial methodological weaknesses and insufficient number of available studies.

## Supporting information

S1 File PRISMA 2009 checklist. (DOC)

S1 Table The excluded studies. (DOCX)
