# Supplementary material for: Patient-reported outcomes of laser-assisted pain control following non-surgical and surgical periodontal therapy: A systematic review and meta-analysis
Source: PLoS One. 2020 Sep 17;15(9):e0238659. doi: 10.1371/journal.pone.0238659 (PMC7498060; doi:10.1371/journal.pone.0238659)
Supplement: S1 Table — (DOCX) [file pone.0238659.s002.docx]

S1Table

The excluded studies
